# Effect of maintaining neck flexion on anti-saccade reaction time: an investigation using transcranial magnetic stimulation to the frontal oculomotor field

**DOI:** 10.1186/1880-6805-32-21

**Published:** 2013-11-13

**Authors:** Kenji Kunita, Katsuo Fujiwara

**Affiliations:** 1 Department of Sports Instruction, Faculty of Sports and Human, Sapporo International University, 4-1-4-1 Kiyota, Kiyota-ku, Sapporo 004-8602, Japan; 2 Department of Human Movement and Health, Graduate School of Medical Science, Kanazawa University, 13-1 Takara-machi, Kanazawa 920-8640, Japan

**Keywords:** Neck flexion, Brain activation, Transcranial magnetic stimulation, Frontal oculomotor field, Anti-saccade reaction time

## Abstract

**Background:**

Reaction time for anti-saccade, in which the gaze is directed to the position opposite to an illuminated target, shortens during maintenance of neck flexion. The present study applied transcranial magnetic stimulation (TMS) to the frontal oculomotor field, and investigated the effect of maintaining neck flexion on information processing time in the anti-saccade neural pathway before the frontal oculomotor field.

**Methods:**

The reaction time was measured with the chin resting on a stand (‘chin-on’ condition) and with voluntary maintenance of neck flexion (‘chin-off’ condition) at 80% maximal neck flexion angle, with and without TMS. The TMS timing producing the longest prolongation of the reaction time was first roughly identified for 10 ms intervals from 0 to 180 ms after the target presentation. Thereafter, TMS timing was set finely at 2 ms intervals from −20 to +20 ms of the 10 ms step that produced the longest prolongation.

**Results:**

The reaction time without TMS was significantly shorter (21.9 ms) for the chin-off (235.9 ± 14.9 ms) than for the chin-on (257.5 ± 17.1 ms) condition. Furthermore, TMS timing producing maximal prolongation of the reaction time was significantly earlier (18.6 ms) for the chin-off than the chin-on condition. The ratio of the forward shift in TMS timing relative to the reduction in reaction time was 87.8%.

**Conclusions:**

We confirmed that information processing time in the anti-saccade neural pathway before the frontal oculomotor field shortened while neck flexion was maintained, and that this reduction time accounted for approximately 88% of the shortening of reaction time.

## Background

In humans, a basic dynamic posture in which the foot, knee, hip, and neck joints, and the trunk are slightly flexed is common for sudden initiation of various motions, and when pursuing a rapidly moving visual target [1]. Maintaining the neck flexion position, which constitutes a part of this dynamic posture, leads to non-specific activation of the brain. This results in: 1) shortened limb and saccade reaction times [2-5]; 2) reduced latencies of visual, auditory, and somatosensory evoked potentials, and increased amplitudes of auditory evoked potentials [6,7]; 3) increased oxy-hemoglobin concentration, taken as an index of cerebral blood flow, in the visual, auditory, and somatosensory areas [6]; 4) increased amplitudes of the event-related potentials associated with motor preparation and cognition [3,8]; and 5) shortened latencies and increased amplitudes of motor evoked potentials (MEPs) evoked by transcranial magnetic stimulation (TMS) [9].

In one experiment, the P100 latency of the visual evoked potential (VEP), reflecting information processing in the neural pathway prior to the visual area, decreased by approximately 3.6 ms while neck flexion position was maintained [7]. By contrast, there was greater shortening in information processing time in the pro-saccade neural pathway beyond the visual area; this decreased by approximately 10 ms during maintenance of the neck flexion position [2]. Irrespective of any differences between information processing associated with the P100 component of the VEP and that associated with the saccade, the fact that shortening of the processing times differed between these two functions suggests that there should be shortening of information processing time beyond the visual area when neck flexion is maintained.

The shortening of reaction time associated with maintaining neck flexion was also found in anti-saccade, in which gaze is oriented to a location situated at the same visual angle but in the opposite direction to an illuminated target [10], and this shortening was of a greater magnitude than that seen in pro-saccade [4]. Multiple cortical and subcortical regions are involved in anti-saccade, including the visual, parietal, prefrontal, and anterior cingulate cortices, frontal and supplementary eye fields, basal ganglia, cerebellum, thalamus, and superior colliculus [11-14]. The frontal eye field (FEF) in the frontal oculomotor field has an important role in saccadic triggering. It is known that TMS interferes with the information processing at the FEF, and that subsequently, the information processing time in the neural pathway involving the FEF is prolonged. In previous studies [15-17], when TMS was applied to the FEF approximately 100 ms after the target presentation, the anti-saccade reaction time increased. Thus, information processing time in the anti-saccade neural pathway before the frontal oculomotor field can be examined by the TMS interference method. Furthermore, it is possible to investigate the shortening of the information processing time beyond the frontal oculomotor field in the anti-saccade neural pathway, based on the reduction in the anti-saccade reaction time and the information processing time occurring before the frontal oculomotor field.

We investigated changes in TMS timing at which maximal prolongation of anti-saccade reaction time was observed during maintenance of neck flexion.

## Methods

### Participants

In a preliminary experiment, we measured the anti-saccade reaction time during maintenance of neck flexion in 14 participants, and selected 11 participants (8 men, 3 women, age (mean ± SD) 23.3 ± 2.9 years) who exhibited shortening of this reaction time. Participants had no history of neurological or orthopedic impairment.

In accordance with the Declaration of Helsinki, all participants provided written informed consent after receiving an explanation of the experimental protocols and how their privacy would be protected. The experimental protocols were approved by our institutional ethics committee.

### Apparatus and data recording

**Figure 1:**
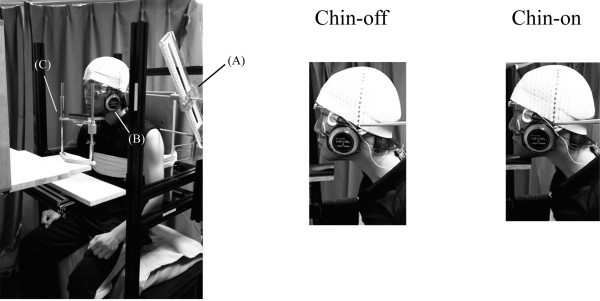
**Experimental setup. (A)** Angle detector for neck angle; **(B)** angle detector for head inclination angle; **(C)** chin stand.

The experimental setup is shown in Figure 1. Participants sat on a steel-frame chair with the back resting against a vertical wall, and the trunk secured by a cotton band to prevent anteroposterior movement. They kept the knees flexed at approximately 90° and rested the feet on a low table. Neck flexion angle was defined as the rotational angle of the tragus around the acromion in the sagittal plane, with the starting position (0°) being a quiet sitting posture. We determined 80% of maximal neck flexion (neck flexion position) for each subject using a custom-made angular detector with the center point set at the acromion, while regulating the distance between the acromion and tragus. Head inclination angle was determined as the angle between the auriculo-infraorbital line and the gravitational line, and this was maintained at the same angle as the sitting posture to maintain constant sensory inputs from the vestibular organ. An angular detector (Level + angle detector; Mitsutomo, Tokyo, Japan), using the pendulum principle, was attached to the temple to confirm this angle. A chin stand was used to support the head and to allow maximal relaxation of the neck extensor muscles.

A visual stimulator (LPK2000; Electro Design, Chiba, Japan) was used to induce saccadic eye movement. Light-emitting diodes (LEDs), which were located at the central fixation point and at the targets, were illuminated for time periods set by a microcomputer within the stimulator. LEDs were placed at the height of the participant’s nose root, and the distance between the LED at the central fixation point and the nose root was set at 50 cm. The central fixation point was illuminated for a random duration of 2 to 4 seconds, and one of the lateral targets was subsequently illuminated for 1 s. The four lateral targets were located at 5 and 10° to the left and right of the central fixation point, and were presented in random. The target triggering a saccade of 10° to the right (10° target to the left) appeared on 70% of occasions, while the other three targets each appeared on 10% of occasions. To measure the calibration amplitude of the anti-saccade, the central fixation point and the target of 10° to the right were illuminated for a duration of 2 seconds each.

Eye movement was measured using the electro-oculography (EOG) technique. Horizontal EOG was recorded from surface electrodes (P-00-S; Ambu, Ballerup, Denmark) on the outer canthus of each eye, and vertical EOG from the electrodes above and below the left eye. A ground electrode was placed at the center of the forehead. Electrode-input impedance was reduced to less than 10 kΩ. The signal from the electrodes was amplified (×2,000) using a DC amplifier (AN-601G; Nihon Kohden, Tokyo, Japan). To obtain stable EOG traces, recording began at least 20 minutes after placement of the electrodes.

**Figure 2:**
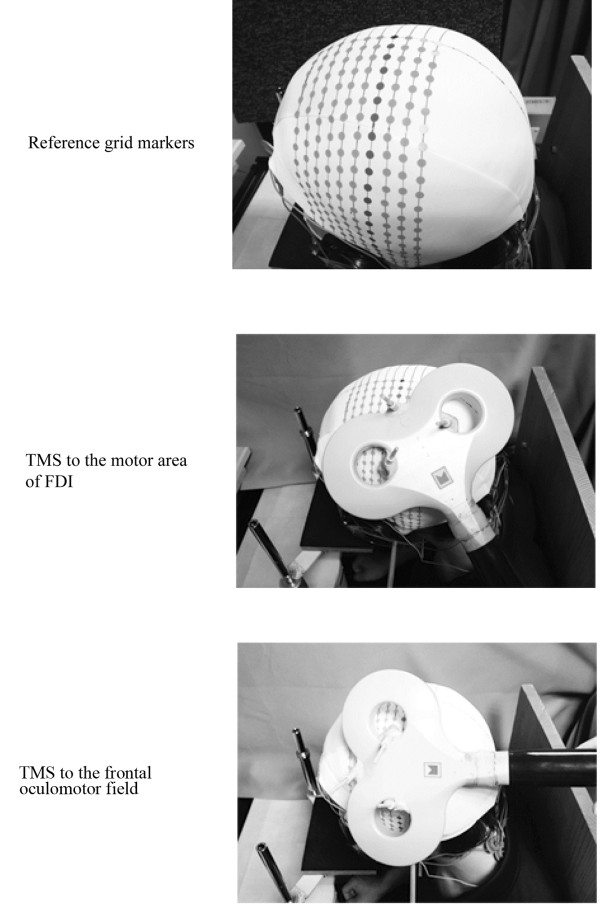
**Reference grid markers and transcranial magnetic stimulation (TMS) to the motor area of the first dorsal interosseous (FDI) muscle and the frontal oculomotor field.**

TMS was applied to the left hemisphere by a magnetic stimulator (MagStim 200, West Wales, UK) with a figure-of-eight coil (each wing 90 mm in diameter). In previous studies, single-pulse TMS has been used to identify the motor area of the first dorsal interosseous (FDI) muscle [18-20] and the frontal oculomotor field [15-17,21]. In the present study, the single-pulse TMS was delivered over various regions of the scalp to identify that motor area and the frontal oculomotor field (Figure 2). The nasion, inion, vertex, and bilateral preauricular points were located according to the 10–20 international electrode method. Reference lines were drawn between the nasion and inion and between the vertex and preauricular points, on a tightly fitting rubber cap. Then grid markers on the left hemisphere were made by drawing additional lines parallel to the reference lines such that the distance between these parallel lines was 1 cm [22]. The hand motor area has been used as a reference point to the areas where saccades were most affected by TMS [16,17,23]. According to previous studies, when applying TMS to the motor area of the FDI muscle, the coil was placed on the scalp with its handle oriented backward and at 45° leftward relative to the midline [19,24,25]. Furthermore, in previous studies, stimulation over the frontal oculomotor field has been performed with the coil handle pointing backwards [21,23,26]. In the present study, the coil handle directions in TMS to the motor area of the FDI muscle and the frontal oculomotor field were consistent with those in previous studies.

Surface electrodes with bipolar derivation were used to monitor and record surface electromyography (EMG) activity of the muscles of the right FDI muscle and the bilateral upper trapezius. Inter-electrode distance was about 1.5 cm for FDI muscle and about 3 cm for trapezius. Signals from electrodes were amplified 1,000 times and band-pass filtered at 20 to 500 Hz for FDI, and 2,000 times and 1.6 to 500 Hz for trapezius, using an EMG amplifier (6R12; NEC, Tokyo, Japan). To monitor the EMG of the trapezius, the signal was directed to a digital oscilloscope (DS-6612; Iwatsu, Tokyo, Japan). Electrode impedances were reduced to less than 2 kΩ.

### Procedure

Prior to the start of measurement, participants contracted and relaxed the shoulder girdle elevator muscles several times, and then exhaled deeply to relax the trapezius muscle. The experimenter verbally instructed the subject to relax the trapezius muscle, and relaxation of that muscle was confirmed by EMG.

**Figure 3:**
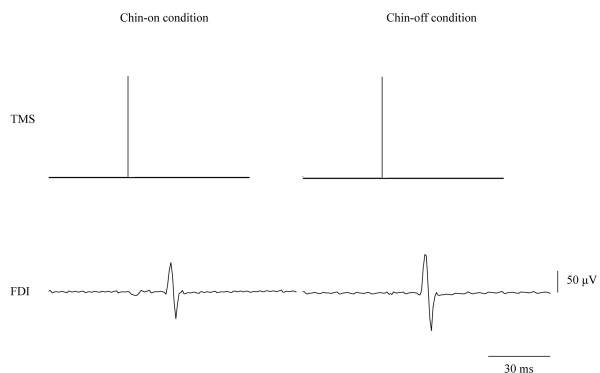
**Motor evoked potential generated by transcranial magnetic stimulation (TMS) to the motor area of the first dorsal interosseous (FDI) muscle on the right side in the first experiment.**

The motor area of FDI was determined as the site at which MEP was elicited by TMS (Figure 3). The minimal TMS intensity required to obtain EMG responses in the FDI muscle exceeding 50 μV in more than half of the trials was used [20,27-29]. Previous studies have reported that the motor area of the FDI muscle is located approximately 5 cm lateral to the vertex [30,31]. Based on the point 5 cm lateral to the vertex, the center of the coil was set at various points in 0.5 cm steps in the anterior-posterior and left-right directions. The position of the FEF in the frontal oculomotor field has been reported at 2 cm anterior or 2 to 4 cm anterior/2 to 4 cm lateral to the hand motor area [16,17,23]. To identify the location of the frontal oculomotor field, based on a location 2 cm anterior to the motor area of the FDI, the TMS coil was positioned at various points in steps of 0.5 cm in the anterior-posterior and left-right directions. We determined in advance the position of the frontal oculomotor field at which TMS induced prolongation of the anti-saccade reaction time. The TMS intensity delivered to the frontal oculomotor field was set 10% above the motor threshold over the motor area of the FDI muscle [21,26,30]. Furthermore, based on a previous study, TMS timing to identify the position of the frontal oculomotor field was set at 80 to 120 ms after target presentation [17].

**Figure 4:**
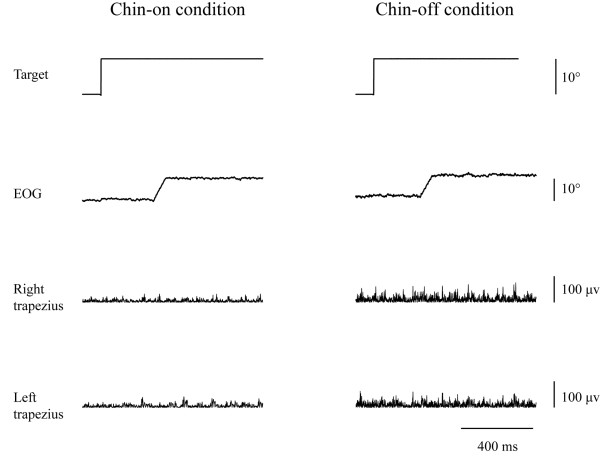
**Representative waves of visual target, electro**-**oculography (EOG), and electromyography of the bilateral upper trapezius.**

**Figure 5:**
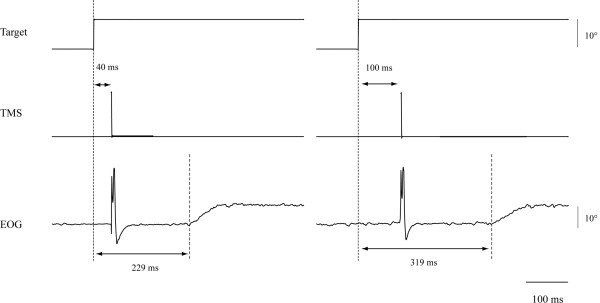
**Typical example generated the interference to information processing associated with the anti-saccade when the transcranial magnetic stimulation (TMS) was applied to the frontal oculomotor field 100 ms after the target stimulation.** EOG, electro-oculography.

**Figure 6:**
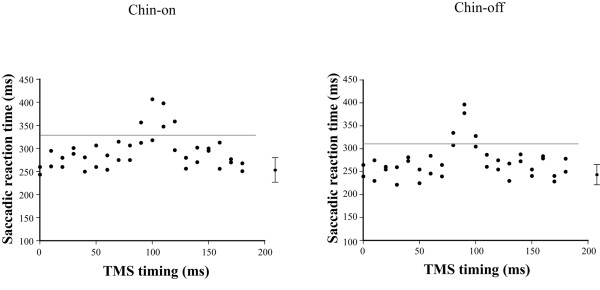
**Typical example of the relationship between application timing of transcranial magnetic stimulation (TMS) and anti-saccade reaction time in the second experiment.**

After identifying the motor area of the FDI muscle and the frontal oculomotor field, the anti-saccade reaction time was measured with the chin resting on a stand (chin-on condition) and with voluntary maintenance of neck flexion (chin-off condition) at 80% maximal neck flexion angle, with and without TMS. First, the reaction time without TMS was measured (first experiment; Figure 4). A trial block under each postural condition comprised 20 trials triggering 10° of saccade to the right. In the second experiment, we varied the TMS timing by 10-ms intervals from 0 to 180 ms after the target presentation (Figure 5). The TMS timing was set at random for each postural condition, and two trials triggering 10° of saccade to the right were obtained for each time period. Total trial numbers in the second experiment were 38 under each postural condition. Figure 6 shows a typical example of the relationship between TMS timing and anti-saccade reaction time. The TMS time period generating the longest prolongation of the reaction time was identified in each condition. In the third experiment, for a −20- to +20 ms bandwidth of the 10 ms step producing the longest prolongation in each condition, TMS timing was set at every 2 ms. The TMS timing was set at random for each condition, and two trials triggering 10° of saccade to the right were obtained for each time period. Total trial numbers in the third experiment were 42 under each postural condition.

The measurement sequence was randomly set between postural conditions for all experiments. Furthermore, after the measurement of saccadic reaction time in each postural condition, the subject alternately gazed at the central fixation point and the target of 10° to the right, and 10 saccadic calibration amplitudes were recorded. A 3 minute rest was taken between each postural condition. Measurements were completed within 2 hours to prevent subject fatigue.

### Data analysis

EOG and visual stimulus data were sent to a computer via an A/D converter at 1,000 Hz with 16-bit resolution. We analyzed the reaction time and the amplitude ratio of practice to calibrated saccades for anti-saccade of 10° to the right. Reaction time was defined as latency to the beginning of eye movement following target appearance. Onset of eye movement was determined by visual inspection of EOG displacement, which was easily discernible from baseline. The saccadic amplitude was determined as the difference of amplitude between 1 second before the onset of the visual target and 1 second after the cessation of saccade. Analysis of saccadic parameters was performed using BIMUTAS-II software (Kissei Comtec, Nagano, Japan).

### Statistical analysis

The Shapiro-Wilks test confirmed that all data satisfied the assumption of normality. The effect of condition on saccadic reaction time, interval between target presentation and TMS application that produced the longest reaction time (target-TMS interval), and amplitude ratio was analyzed using a paired *t*-test. Pearson’s correlation was used to examine the relationship between the shortening of the target-TMS interval and the reduction in anti-saccade reaction time. Alpha level was set at *P* = 0.05. All statistical analyses were performed using SPSS 14.0 J (IBM Japan, Tokyo, Japan). All data are presented as mean ± SD.

## Results

The motor area of FDI was located 3.6 ± 1.0 cm lateral to the vertex and 0.3 ± 0.7 cm anterior to the line between the vertex and preauricular points. The TMS intensity at this point was 54.0 ± 6.2%. The position of the frontal oculomotor field at which prolongation of reaction time by TMS was observed was 4.9 ± 1.2 cm lateral to the vertex, and 2.5 ± 1.1 cm anterior to the line between the vertex and preauricular points. The TMS intensity at this point was 59.3 ± 7.3%. The position of the frontal oculomotor field was 1.1 ± 1.0 cm lateral and 2.4 ± 0.7 cm anterior to the motor area of FDI.

**Figure 7:**
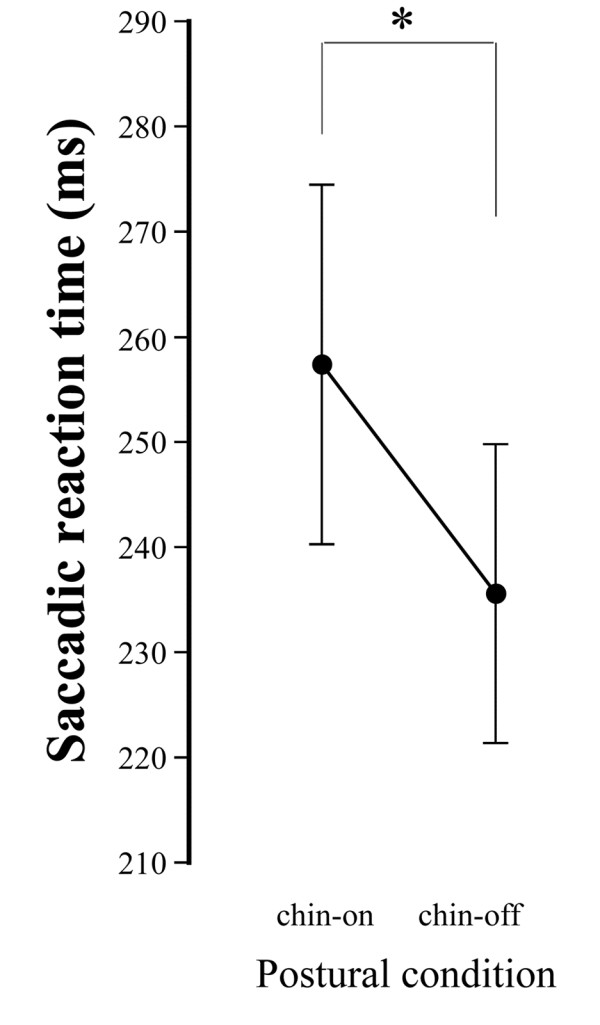
**Mean and standard deviation of anti-saccade reaction time without transcranial magnetic stimulation under the chin-on and chin-off conditions.** *P < 0.01.

**Figure 8:**
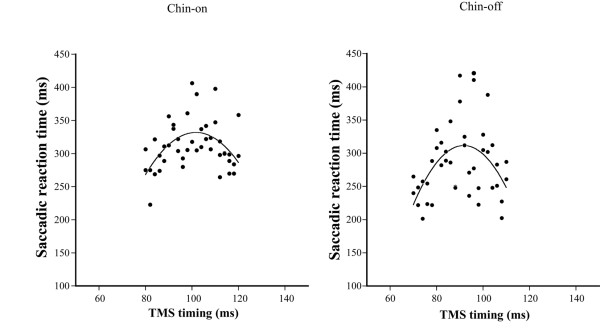
**Typical example of the relationship between application timing of transcranial magnetic stimulation (TMS) and anti-saccade reaction time in the third experiment.**

**Figure 9:**
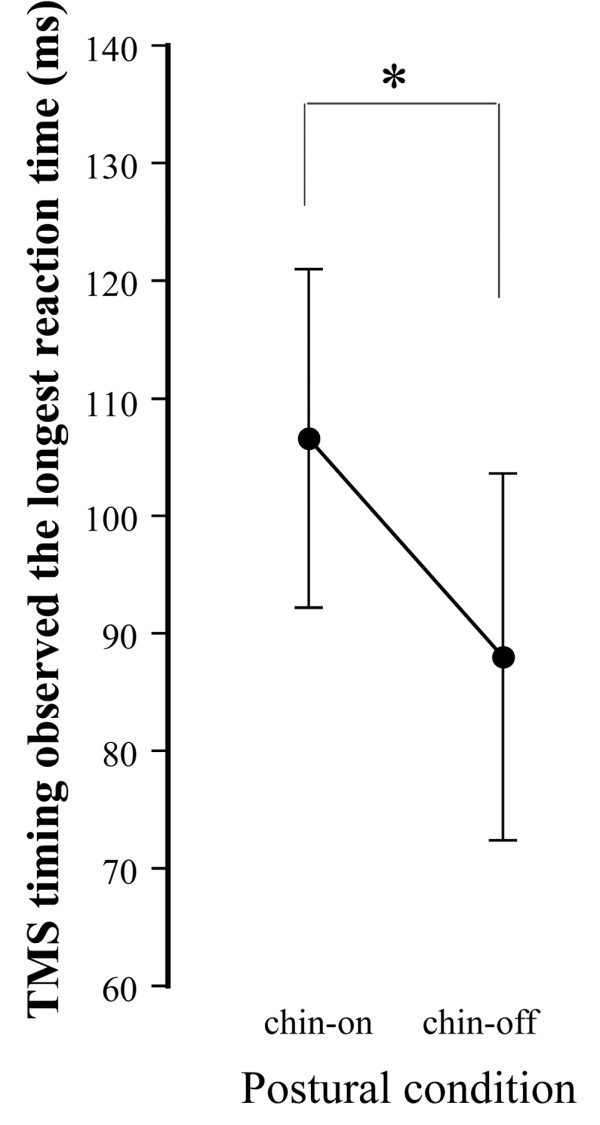
**Transcranial magnetic stimulation (TMS) timing producing the longest anti-saccade reaction time under the chin**-**on and chin**-**off conditions.** Data are mean and standard deviation. *P < 0.01

**Figure 10:**
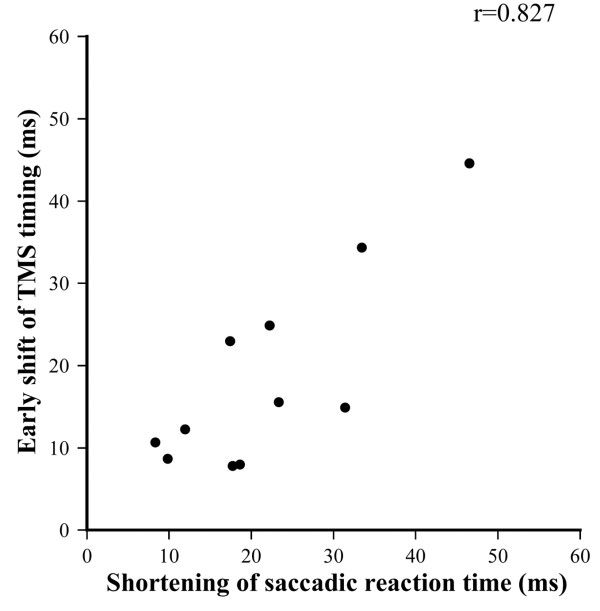
**Correlation of early shifts in timing of transcranial magnetic stimulation (TMS) that produced the longest reaction time and reduction in the anti-saccade reaction time, under the chin-off condition compared with the chin-on condition.**

Amplitude ratio of practice to calibrated-saccades ranged from 99.1 ± 29.9% to 105.3 ± 22.9% in the first and third experiments. Figure 7 shows the anti-saccade reaction time without TMS under both conditions. The reaction time was significantly shorter under the chin-off condition than under the chin-on condition (235.9 ± 14.9 ms vs. 257.5 ± 17.1 ms, degrees of freedom (df) = 10, *t* = 6.33, *P* < 0.01), representing a shortening of 21.9 ± 11.4 ms. A typical example of the relationship between TMS timing and anti-saccade reaction time in the third experiment is shown as a quadratic function in Figure 8. The correlation coefficient between the two parameters, calculated by a quadratic function, ranged from 0.320 to 0.681 under the chin-on condition, and from 0.312 to 0.644 under the chin-off condition, hence the two parameters were found to be significantly correlated. The TMS timing eliciting the longest reaction time, estimated by the quadratic function, was significantly shorter under the chin-off condition than under the chin-on condition (88.0 ± 15.6 ms vs. 106.6 ± 14.4 ms, df = 10, *t* = 5.16, *P* < 0.01; Figure 9). This represented a shift toward earlier TMS timing of 18.6 ± 12.0 ms. The correlation coefficient between the shortening of the target-TMS interval and aforementioned shortening of the anti-saccade reaction time was significant at 0.827 (df = 9, *t* = 4.41, *P* < 0.01; Figure 10). The ratio of the early shift in TMS timing relative to shortening of the reaction time was 87.8 ± 32.8%.

## Discussion

A previous study found that saccadic reaction time varied with saccade amplitude [32]. In the present study, amplitude ratio of practice to calibrated saccades ranged from 99.1% to 105.3% in the first and third experiments. Thus, the error in amplitude ratio was less than 10%, indicating that the error in movement angle was less than 1°. In the previous study, when the error in visual angle was less than 1°, no difference in saccadic reaction time according to visual angle was observed [32]. We can accordingly discuss the different effects of the chin-on and chin-off conditions on saccade reaction time and the TMS timing that produced the longest reaction time, without addressing the effect of eye movement amplitude.

The frontal oculomotor fields related to anti-saccade are the FEF and the prefrontal cortex. TMS applied to the FEF, which is involved in saccadic triggering, has been reported to interfere with saccadic reaction time [16,17]. Terao *et al*. found that the anti-saccade reaction time lengthened when TMS was applied to the FEF at 100 ms after target presentation [17]. In the present study, the TMS timing that had the greatest effect on anti-saccade reaction time was 106.6 ± 14.4 ms in the chin-on condition, consistent with that previous study [17]. The position of the FEF in the frontal oculomotor field has been reported at 2 cm anterior or 2 to 4 cm anterior/2 to 4 cm lateral to the hand motor area [16,17,23]. The position of the FEF in relation to the motor area of FDI, based on Talairach coordination in functional magnetic resonance imaging studies, is consistent with its position in TMS studies [12,23,26,33]. In the present study, the position of the frontal oculomotor field observed to prolong the anti-saccade reaction time was located 2.4 cm anterior and 1.1 cm lateral relative to the motor area of FDI. This therefore strongly suggested that our stimulating position corresponded to the FEF.

The important finding of the present study was that the interval between target presentation and TMS application generating the longest reaction time was significantly shorter under the chin-off than the chin-on condition. The present study is the first in which information processing time in the anti-saccade neural pathway before the frontal oculomotor field decreased during voluntary maintenance of the neck flexion position. This suggests that information processing speed in the saccade pathway before the frontal oculomotor field was markedly increased by the brain activation associated with maintaining the neck flexion position. In the neck flexion position, the neck extensors activate to maintain the head and neck in the gravitational environment. The brain activation is presumably due to ascending activation associated with muscular sensory information from the neck extensors, and/or descending activation from the cerebral cortex, which includes attention-related processes [34-38]. In a previous study, saccadic reaction time decreased during vibration of the trapezius muscle when the neck was in a resting position [39]. This finding supports the existence of ascending brain activation from the neck extensors. Morruzi and Magoun [40] proposed that the ascending brain activation system originates at the brainstem reticular formation, and the system has since been examined using animal studies, pharmacological experiments, and neurological treatments for patients with brain dysfunction [38,41,42]. Currently, the activation system is known to consist of two subsystems: a dorsal pathway from the reticular formation to the thalamus and cortex, and a ventral pathway from the reticular formation to the hypothalamus and cortex [41,42].

The correlation between shortening of the target-TMS interval and reduction in the reaction time during maintenance of the neck flexion position was significant (*r* = 0.827). The ratio of shortening of the target-TMS interval to reduction in the reaction time was 87.8%. Hence, when neck flexion was maintained, reduction in information processing time before the frontal oculomotor field accounted for approximately 88% of the shortening of anti-saccade reaction time. The present study could not identify which portion of the neural pathway was involved in this reduction in reaction time. However, findings relevant to this have been reported previously. In one study, the P100 latency of the VEP decreased by approximately 3.6 ms when participants maintained the neck flexion position [7]. Another study showed that the P100 component reflects sensory information processing in the calcarine sulcus within the visual area [43]. Although the information processing associated with the P100 component of the VEP differs from that associated with anti-saccade, the time reduction in the pathway before the frontal oculomotor field was 18.6 ± 12.0 ms in anti-saccade, five times greater than the reduction in P100 latency. This suggests that cortical information processing from the visual area to the frontal oculomotor field is greatly affected by the brain activation associated with maintaining the neck flexion position. By contrast, the neural pathway from the frontal oculomotor field to the extraocular muscle encompasses the basal ganglia, superior colliculus, and abducens nerve. The information processing time on this neural pathway beyond the frontal oculomotor field, based on the anti-saccade reaction time and timing of TMS that induced prolongation of the reaction time, was approximately 150 ms, accounting for the three-fifths of the anti-saccade reaction time. However, the reduction in information processing time on the neural pathway beyond the frontal oculomotor field associated with maintaining neck flexion was approximately 3 ms, much smaller than that on the neural pathway before the frontal oculomotor field.

## Conclusions

It was clear that the information processing time in the anti-saccade neural pathway before the frontal oculomotor field shortened by 18.6 ms (average) with voluntary maintenance of neck flexion. This time accounted for approximately 88% of the shortening of anti-saccade reaction time.

## Abbreviations

A/D: Analog-to-digital; df: degrees of freedom; EMG: Electromyography; EOG: Electro-oculography; FDI: First dorsal interosseous; FEF: Frontal eye field; LEDs: Light-emitting diodes; MEPs: Motor evoked potentials; TMS: Transcranial magnetic stimulation; VEP: Visual evoked potentials.

## Competing interests

The authors have no competing interests to disclose.

## Authors’ contributions

Contribution of each author is as follows: KK presented all the idea of this study, planed the method, directed the experiments, data analyses and interpreted the results. Sentences in Introduction and Discussion including Conclusion were written by KK and KF. All authors read and approved the final manuscript.
